# A novel murine model for assessing fetal and birth outcomes following transgestational maternal malaria infection

**DOI:** 10.1038/s41598-019-55588-8

**Published:** 2019-12-20

**Authors:** Catherine D. Morffy Smith, Brittany N. Russ, Alicer K. Andrew, Caitlin A. Cooper, Julie M. Moore

**Affiliations:** 1 Department of Infectious Diseases and Center for Tropical and Emerging Global Diseases, University of Georgia, Athens, GA United States; 2 Department of Infectious Diseases and Immunology, University of Florida, Gainesville, FL United States

**Keywords:** Animal disease models, Malaria

## Abstract

*Plasmodium falciparum* infection during pregnancy is a major cause of severe maternal illness and neonatal mortality. Mouse models are important for the study of gestational malaria pathogenesis. When infected with *Plasmodium chabaudi chabaudi* AS in early gestation, several inbred mouse strains abort at midgestation. We report here that outbred Swiss Webster mice infected with *P*. *chabaudi chabaudi* AS in early gestation carry their pregnancies to term despite high parasite burden and malarial hemozoin accumulation in the placenta at midgestation, with the latter associated with induction of heme oxygenase 1 expression. Infection yields reduced fetal weight and viability at term and a reduction in pup number at weaning, but does not influence postnatal growth prior to weaning. This novel model allows for the exploration of malaria infection throughout pregnancy, modeling chronic infections observed in pregnant women prior to the birth of underweight infants and enabling the production of progeny exposed to malaria *in utero*, which is critical for understanding the postnatal repercussions of gestational malaria. The use of outbred mice allows for the exploration of gestational malaria in a genetically diverse model system, better recapitulating the diversity of infection responses observed in human populations.

## Introduction

Gestational *Plasmodium falciparum* infection results in severe maternal illness and an array of poor pregnancy outcomes, including low birth weight^1–6^. Reports of poor birth outcomes associated with *P*. *vivax* infection are raising awareness that this infection also poses a risk for maternofetal health (as reviewed by Rogerson, 2018)^7^. In the absence of malaria control programs targeting pregnant women, an estimated 9.5 million pregnant women would have been exposed to malaria in 2015 and 750,000 low birthweight babies would have been born as a result^7,8^. Intermittent preventative treatment in pregnancy protected an estimated 128,000 infants from this outcome^7,8^; however, gestational malaria remains a significant cause of infant death with an estimated mortality rate of 37.5% for malaria-associated low birth weight^9^. In addition to an increased risk of death, children born to women infected with malaria during their pregnancies can face an increased risk of developing neurodevelopmental disorders^10^ and clinical malaria in early life^11–13^. Therefore, preventing and treating malaria in pregnant women remains an urgent public health challenge because the negative impact of maternal malaria infection on child health and development extends beyond the perinatal period.

The poor birth outcomes associated with human gestational *P*. *falciparum* malaria are associated with accumulation of parasite-infected red blood cells (iRBCs) within the intervillous spaces of the placenta. iRBC sequestration in the placenta is mediated by the expression of the VAR2CSA variant of *P*. *falciparum* erythrocyte membrane protein 1 (PfEMP1), which allows the iRBCs to bind to chondroitin sulfate A (CSA)-bearing proteoglycans expressed by the syncytiotrophoblast^14–16^. VAR2CSA-mediated placental sequestration of iRBCs is a unique feature of *P*. *falciparum* infection; however, infection with other *Plasmodium* species, specifically *Plasmodium vivax*, can also result in severe maternal illness, placental pathology^17^, and low birth weight^3^. Limited studies suggest that *P*. *vivax* may also exhibit limited ability to bind to placental CSA^18,19^. A number of pathologies have been extensively described in the *P*. *falciparum*-infected placenta, including intervillositis^20,21^, lipid peroxidation^22^, fibrin deposition^23–25^, syncytial knotting^23,24,26^, and villous tissue necrosis^24^. These pathological features are associated with the disruption of a number of placental processes and functions, including placental perfusion^27^, amino acid transport^28,29^, glucose transport^30,31^, and autophagy^32^, which limit the placenta’s ability to support the developing fetus. Similar placental changes may also occur in *P*. *vivax* infection^33^. The etiology of such pathologies remains to be determined but suggests that malaria pathogenesis in pregnancy is complex and multifactorial and intimates that specific and extensive parasite sequestration in the placenta may not be absolutely required for placental dysfunction and poor birth outcomes in gestational malaria.

Mouse models for malaria infection in pregnancy allow for the interrogation of these mechanisms in an inexpensive, genetically tractable model system. Different mouse models best recapitulate different features of gestational malaria. Infection with *Plasmodium berghei* ANKA or *P*. *berghei* NK65 recreate the placental accumulation of iRBCs and maternal inflammatory infiltrate^34^, commonly observed in human malaria at term^14^. *P*. *berghei* interacts with CSA in the murine placenta^34^, and recrudescent infections in pregnant animals are associated with enhanced capacity to interact with this glycan in the placenta^35^.When initiated in early gestation, *P*. *berghei* infection results in abortion and embryo resorption^36,37^. Infection initiated in mid- to late-gestation causes preterm delivery, low birthweight, and stillbirth^36–38^. Despite the diversity of possible pregnancy outcomes, *P*. *berghei*-based models are limited by the extreme virulence and rapid progression of *P*. *berghei* infection; even with recrudescent infections, wherein parasitemia becomes patent after mid-gestation, one third of dams succumb to the infection^35^. As a result, *P*. *berghei*-based models cannot be used to study prolonged infection over the course of gestation. In contrast, infection with *Plasmodium chabaudi chabaudi* AS is not lethal in C57BL/6 (B6) mice. Although not formally demonstrated to interact with CSA, *P*. *chabaudi* is a cytoadherent parasite^39,40^. Infection of B6 mice on gestational day (GD) 0 results in midgestational pregnancy loss associated with the accumulation of iRBCs in the placenta and the disruption of placental architecture^41–46^. This infection may better model the trajectory of human infection in pregnancy, as dams develop severe maternal illness but survive.

While pregnancy loss is observed in a small subset of malaria-infected pregnant women, those residing in malaria-endemic regions often develop chronic infections but carry their pregnancies to term^47^. The current mouse models for gestational malaria are limited in scope because they do not model persistent infection over the course of gestation culminating in a live birth. In addition, most available models utilize inbred mice, which limit the generalizability of the results to genetically diverse human populations. To overcome these limitations, we have developed a novel model for pregnancy maintenance during maternal malaria infection utilizing outbred Swiss Webster mice. Following infection with *P*. *chabaudi chabaudi* AS on GD 0, these mice exhibit reduced fetal weight and viability at term, and infected dams have fewer pups at weaning, although growth to weaning is normal for surviving pups. Stasis in weight gain at midgestation is associated with heavy parasite burdens, significant hemozoin accumulation, and an associated heme oxygenase response in placentae. This novel model will allow for the exploration of the complex host-parasite interactions that cause significant maternal morbidity and placental damage but allow for fetal survival. In addition, this model enables the straightforward production of progeny exposed to malaria infection and malaria-induced maternal responses *in utero*, facilitating exploration of prenatal exposure to malaria on postnatal development, which is critical because gestational malaria remains a leading cause of preventable infant death^48^.

## Results

### Swiss Webster mice maintain pregnancies to term despite developing high parasite burdens and severe malarial anaemia

**Figure 1:**
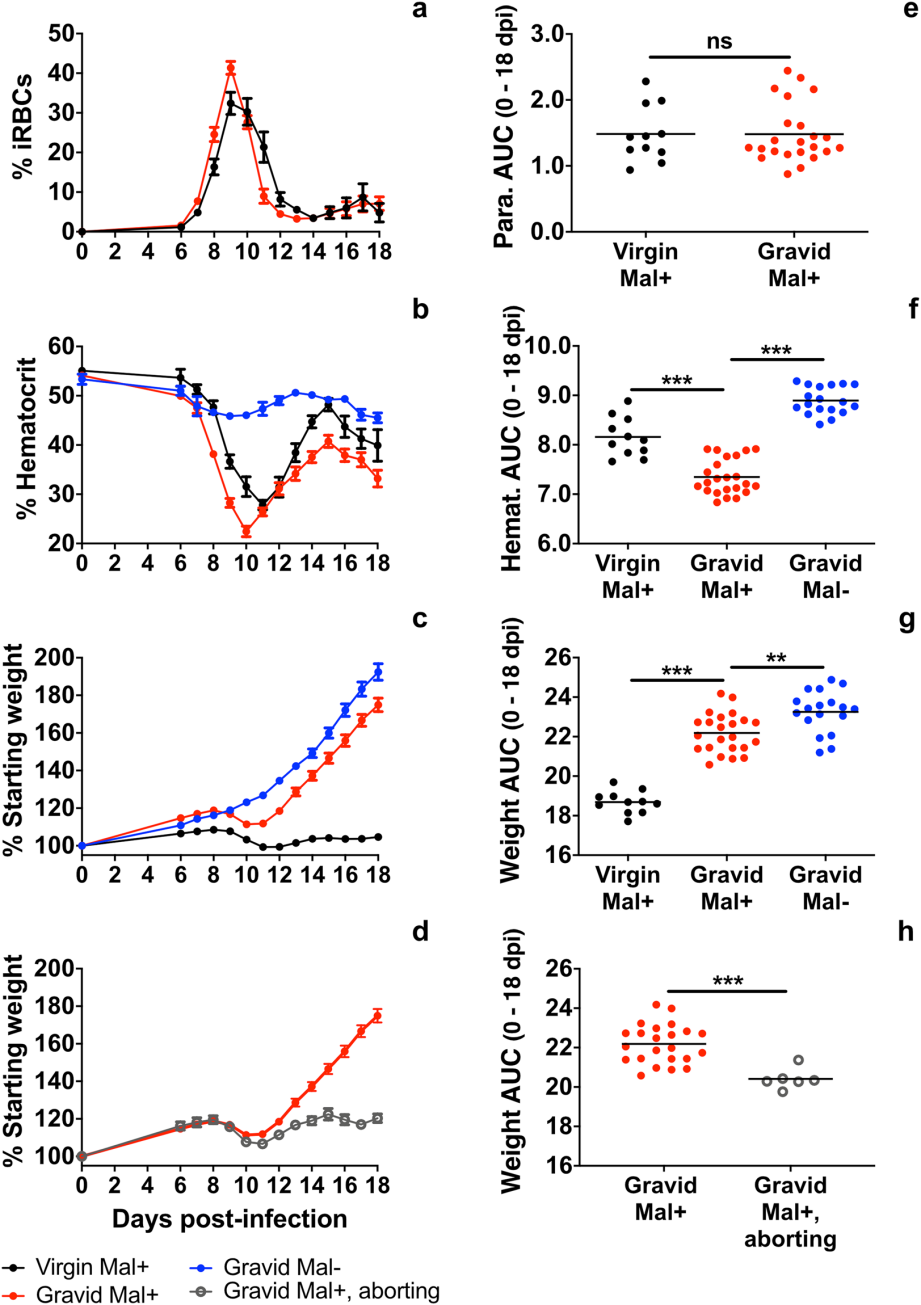
Parasitemia, hematocrit, and weight change in *P*. *chabaudi chabaudi* AS-infected Swiss Webster mice. Course of infection data for virgin Mal+ mice, gravid Mal+, and gravid Mal− mice are presented. The gravid Mal+ group only contains dams that carried pregnancies to term. Gravid Mal+ mice that underwent spontaneous abortion are labeled ‘gravid Mal+, aborting,’ and are depicted in panels (d and h). (**a**) Parasitemia in virgin and gravid Mal+ mice was estimated by flow cytometry and is presented as the percentage of iRBCs in the peripheral blood. (**b**) Percent hematocrit was measured throughout infection in virgin and gravid Mal+ mice and gravid Mal− controls. (**c**) Weight change in virgin and gravid Mal+ mice and gravid Mal− mice is presented as the percentage of body weight relative to 0 days post-infection or post-mock infection. (**d**) Weight change in gravid Mal+ mice that aborted their pregnancies at midgestation is presented as the percentage of body weight relative to 0 days post-infection or post-mock infection. The curve depicting weight change in gravid Mal+ mice that carry their pregnancies to term is duplicated in this panel for comparison. (**e**) AUC was calculated for the parasitemia curve of each individual mouse. A statistically significant difference in parasite burdens is not detected between virgin and gravid Mal+ mice (Student’s *t* test, *P* = 0.9355). (**f**) AUC was calculated for the hematocrit curve of each individual mouse. Statistically significant differences in hematocrit are observed between virgin and gravid Mal+ mice (*P* < 0.001) and between gravid Mal− and Mal+ mice (*P* < 0.001; one-way ANOVA with Bonferroni multiple group comparisons). (**g**) AUC was calculated for the weight change curve of each individual mouse. Statistically significant differences in weight change are observed between gravid Mal+ and Mal− mice (*P* < 0.01), as well as virgin and gravid Mal+ mice (*P* < 0.001; one-way ANOVA with Bonferroni multiple group comparisons). (**h**) AUC was calculated for the weight change curve of each individual mouse. A statistically significant difference in weight change is detected between aborting and non-aborting gravid Mal+ mice (*P* < 0.0001; Mann-Whitney U test). The AUC values for gravid Mal+ mice that carry their pregnancies to term are duplicated in this panel for comparison. Virgin Mal+ *n* = 11; Gravid Mal+ *n* = 23; Gravid Mal− *n* = 18; Gravid Mal+, aborting *n* = 6; ***P ≤ 0.001; **P ≤ 0.01; NS,*P* > 0.05.

Swiss Webster mice are highly susceptible to *P*. *chabaudi chabaudi* AS infection; in this study, infection was initiated on GD 0, or experimental day (ED) 0. Between GD/ED 6 and GD/ED 18, parasitemia was estimated daily by flow cytometry for each malaria-infected (Mal+) mouse. Both virgin and gravid Swiss Webster mice developed high peripheral parasite burdens, with most animals achieving peak infection on GD/ED 9 or GD/ED 10 (Fig. 1a). Among virgin mice, 7/13 (54%) achieved a second peak of parasitemia (greater than 2% above the nadir), a pattern mirrored by gravid mice (16/25, 64%; *P* > 0.05 by two-tailed Fisher’s exact test). There was likewise no significant difference in parasite burden between virgin Mal+ and gravid Mal+ mice over the course of infection (Fig. 1e) and amplitude of recrudescent infection was also similar (mean +/- SD: 17.0 ± 3.9% and 14.3 ± 10.8%, respectively; *P* > 0.05 by Mann-Whitney U test). No fatalities were observed as a result of infection.

Malarial anaemia, a major cause of morbidity and mortality in malaria-infected humans and rodents, is an important indicator of infection severity and morbidity in pregnancy^5,41,49,50^. Infection-related anaemia, indicated by reduced hematocrit, was most severe in the one to two days following the development of peak parasitemia, GD/ED 10 and GD/ED 11 (Fig. 1b). A subtle reduction in hematocrit was observed in gravid uninfected (Mal−) mice over the course of gestation (Fig. 1b), likely due to the increase in blood volume observed in pregnant animals^51–53^. Infection in gravid mice was associated with a more significant reduction in hematocrit over the course of gestation than was observed in virgin Mal+ mice (Fig. 1f), likely reflecting the cumulative impact of malaria-associated anaemia and pregnancy-related hemodilution^51–53^.

Unlike *P*. *chabaudi chabaudi* AS-infected B6 or A/J mice^41–45,54^, Swiss Webster mice infected on GD 0 did not uniformly abort their pregnancies at midgestation. In mated female mice, pregnancy was indicated by an increase in body weight of at least 10% by GD 8 (Supp. Fig. 1). Six of 29 (21%) Mal+ females that met this cut-off did not produce viable fetuses at term, indicative of spontaneous pregnancy loss. Such pregnancy loss was not observed in any gravid Mal− mice. Between GD/ED 9 and GD/ED 11, non-aborting gravid Mal+ mice displayed a stasis in weight gain compared to gravid Mal− controls, which gained weight steadily over the course of gestation (Fig. 1c). However, from GD/ED 11 onward, gravid Mal+ and gravid Mal− mice gained weight at approximately the same rate (Fig. 1c), indicating that gravid Mal+ mice remained pregnant and that their fetuses continued to grow as parasite burdens descended (Fig. 1a). Despite this recovery, infection significantly reduced overall pregnancy-related weight gain in gravid mice between GD/ED 0 and GD/ED 18 (Fig. 1g).

Notably, the group of Mal+ dams that experienced spontaneous abortion around the time of peak infection did not develop higher parasite burdens or a more severe anemia than the gravid Mal+ mice that carried pregnancies to term (see Supplementary Data). As expected, weight change over the course of gestation was significantly reduced in aborting Mal+ dams mice compared to Mal+ dams that did not experience pregnancy loss (Fig. 1d,h).

### Maternal infection does not significantly reduce fetal viability at term

To determine litter size, fetal viability, fetal weight, and placental weight prior to parturition, dams were sacrificed at gestational term (GD 18) and uteri were removed for assessment. Fetuses and placentae were dissected away from the uterus and weighed, and fetal viability was determined by reactive movement.

**Figure 2:**
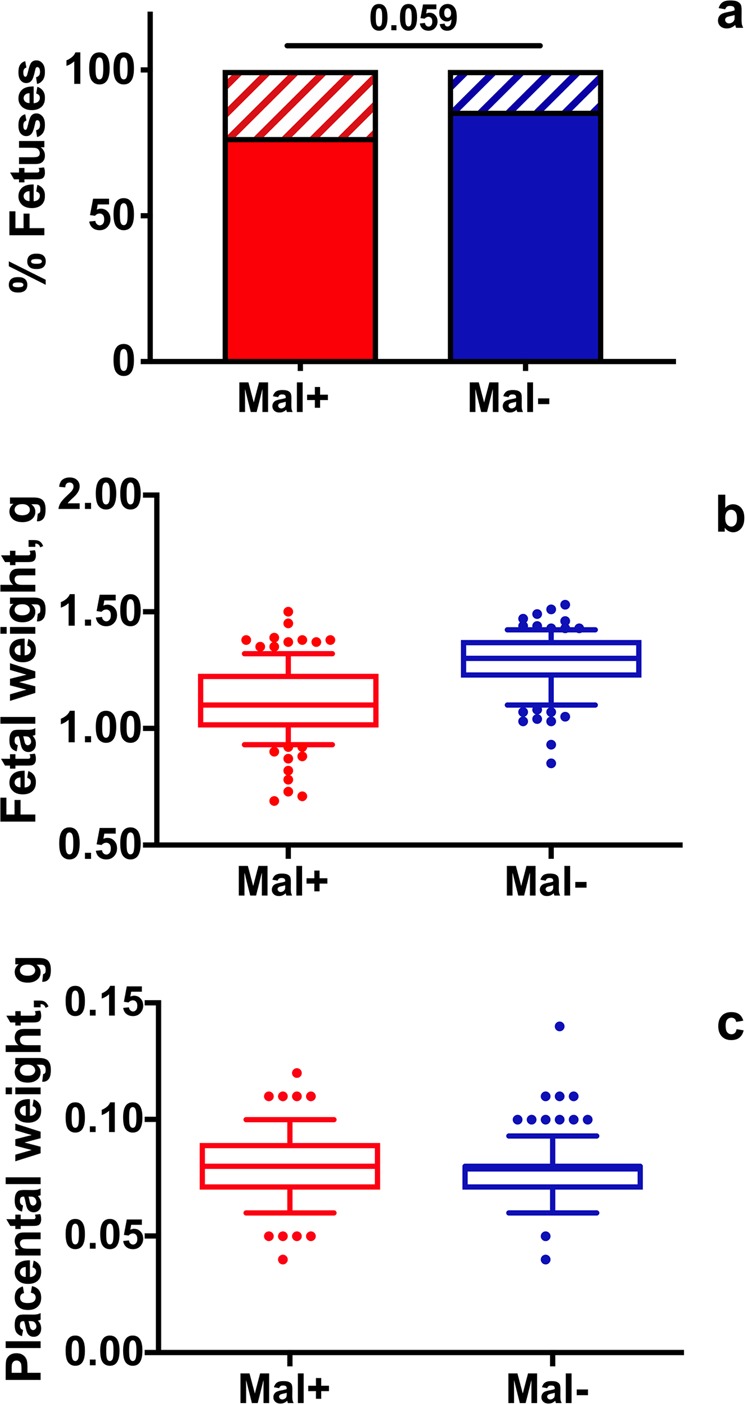
Impact of maternal infection status on fetal number, fetal weight, and placental weight at term. Mal+ and Mal− dams were sacrificed at GD 18 for assessment of pregnancy outcome. Gravid cohorts do not include dams that aborted prior to GD 18. (**a**) Number of viable (solid bars) and nonviable (hatched bars) fetuses are presented as proportions of total fetuses produced by the populations of Mal+ and Mal− dams. Mal+ dams tend to produce a lower proportion of viable fetuses than Mal− controls (*P* = 0.059, two-tailed Fisher exact test). Gravid Mal+ *n* = 109/142 viable/total fetuses, 13 litters; Gravid Mal− *n* = 106/123 viable/total fetuses, 11 litters. (**b**) Weights of viable fetuses pooled by dam infection group are presented for visualization only. Fetal weights were analyzed by mixed linear models. Infection status, starting dam weight, and the number of viable fetuses within a litter account for 46% of the variance in fetal weight between dams (*P* < 0.0001). If dam weight and the number of viable pups is held constant, infection significantly influences fetus weight and is associated with a 0.21 g reduction in fetal weight (*P* = 0.0002). Gravid Mal+ *n* = 109 fetuses, 13 litters; Gravid Mal− *n* = 106 fetuses, 11 litters. (**c**) Weights of placentae associated with viable fetuses, pooled by dam infection group are presented for visualization only. Placental weights were analyzed by mixed linear models. Placental weight was not significantly influenced by infection status, starting dam weight, or litter size. Gravid Mal+ *n* = 109 placentae, 13 litters; Gravid Mal− *n* = 106 placentae, 11 litters.

Infection was not associated with a reduction in the mean number of total fetuses nor the mean number of viable fetuses produced by Mal+ dams relative to Mal− dams. Mal+ dams (*n* = 13) produced a mean of 11 ± 1 fetuses (±SD) at term, while Mal− dams (*n* = 11) produced an average of 11 ± 2 fetuses (*P* > 0.05; Student’s *t* test). The mean number of viable fetuses produced by dams in each cohort was also similar. Mal+ dams produced an average of 8 ± 3 viable fetuses (±SD) per litter, while Mal− dams produced 10 ± 3 fetuses (*P* > 0.05; Student’s *t* test). However, maternal infection was associated with a tendency towards reduced fetal viability at a population level (Fig. 2a). A cohort of 11 Mal− dams produced 123 fetuses, 106 of which were viable (86%). In contrast, a cohort of 13 Mal+ dams produced 142 fetuses, 109 of which were viable (77%; *P* = 0.059; two-tailed Fisher’s exact test).

When fetal and placental weights were pooled by maternal infection status, infection appeared to be associated with a reduction in fetal weight on a population level (Fig. 2b), whereas placental weights were unchanged (Fig. 2c). However, fetal and placental weights varied both within and between dams (Supp. Fig. 2). To determine the impact of maternal malaria infection throughout gestation on fetal and placental growth while controlling for the lack of independence of pups born to the same dam, fetal and placental weights were analyzed by mixed linear models. Infection status, starting dam weight, and the number of viable fetuses within a litter account for 46% of the variance in fetal weight between dams (*P* < 0.0001). If dam weight and the number of viable pups are held constant, infection is associated with a statistically significant 0.21 g reduction in fetal weight (*P* = 0.0002). It is important to note that the variance in body weight observed in Swiss Webster dams randomized to Mal+ or Mal− cohorts was evenly distributed across infection groups at GD/ED 0 (Supp. Fig. 3). Placental weight was not significantly influenced by any of the variables included in the analysis.

### Malaria-infected dams produce fewer live pups at weaning, but maternal infection does not impact pre-weaning pup growth

**Figure 3:**
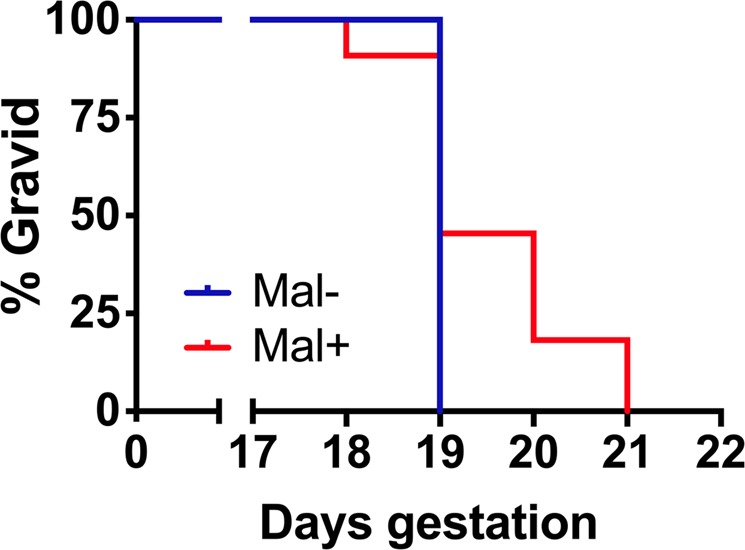
Duration of gestation in *P*. *chabaudi chabaudi* AS-infected and uninfected gravid mice. Infection does not significantly impact the duration of gestation (*P* = 0.1188; Mantel-Cox test). Gravid Mal− *n* = 7; Gravid Mal+ *n* = 11.

To determine the impact of malaria infection on postnatal outcomes, gravid Mal− and Mal+ mice were allowed to proceed to delivery. Gestational duration did not differ significantly as a function of infection, although Mal+ and Mal− cohorts exhibited different patterns (Fig. 3). All gravid Mal− mice spontaneously delivered their litters on GD 19, while gravid Mal+ animals displayed a greater variation in gestational duration, delivering their litters between GD 18 and GD 21. Of the 11 gravid Mal+ mice, two delivered live pups that did not survive to 4 days of age. These litters were delivered on GD 18 and GD 19, respectively, following weight loss between GD 17 and GD 18 of 8.73 and 0.74 g, respectively. The dramatic weight loss in the former might be attributable to the dam weight being captured while the mouse was in the process of delivering, but had already cannibalized pups, as none were evident at that time; later in the day, though, neonates were visible in the cage and noted to be cyanotic and less active relative to normal, healthy neonates. Pup number and exact time of death was not determined for these litters, however, as human disruption of the nest was strenuously avoided in the first 4 days of life to prevent stress- or disruption-induced rejection of the litter.

Malaria infection was associated with a statistically significant reduction in pup number at weaning. Mal− dams (*n* = 7) had a mean of 12 ± 2 pups (±SD) at weaning, while Mal+ dams (*n* = 11) had a mean of 5 ± 4 pups at weaning (*P* = 0.0005, Mann-Whitney U test). The reduction in gravid Mal+ progeny observed at weaning likely occurs between birth and 4 days of age (when weights can be safely measured in newborns) because infection was not associated with a significant reduction in the average number of viable fetuses produced by Mal+ dams at GD 18. Furthermore, the loss of pups between 4 and 22 days of age was a rare event, with only a single pup produced by a Mal+ dam perishing during this period. Intriguingly, Mal+ dams overall produced significantly more male progeny (33/51, 65%) than Mal− dams (39/86, 45%; *P* = 0.032, two-tailed Fisher’s exact test).

**Figure 4:**
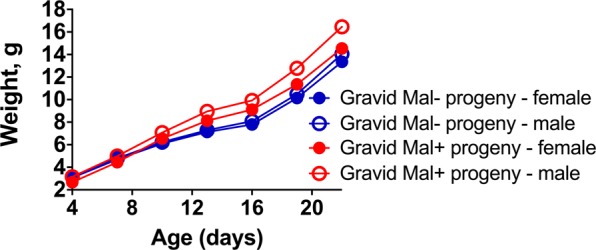
Postnatal pup growth. Weights between 4 and 22 days of age are presented for the progeny of Mal+ and Mal− dams. Gravid Mal− *n* = 7 litters; Gravid Mal+ *n* = 9 litters. Pup numbers per group are as follows: Gravid Mal− female progeny *n* = 47; Gravid Mal− male progeny *n* = 39; Gravid Mal+ female progeny *n* = 18; Gravid Mal+ male progeny *n* = 33. A single pup, produced by a Mal+ dam, perished between 11 and 13 days of age and due to missing data is censored from this analysis.

Individual pup growth was tracked between birth and weaning to assess the impact of infection on postnatal growth and development. Starting at 4 days of age, each pup was weighed every three days until weaning at 22 days of age (Fig. 4). In general, a negative relationship between litter size and the average weight of sibling pups was detected (Supp. Fig. 4); however, within groups, this relationship was only significant for progeny of Mal− dams (Pearson *r* = −0.8969, *P* = 0.0062). Trends in pup growth were analyzed by linear mixed models. No significant relationship between maternal infection status and pup growth trends was detected. Consistent with the observed negative influence of large litter size on pup growth (Supp. Fig. 4), the model revealed that number of pups in the litter at weaning (*P* = 0.0001) significantly influenced the pup growth trajectory. Weight of the dam also exerted an effect that varied over time (interaction term, *P* = 0.0215). Pup sex did not significantly impact weight change over time.

### Infection reduces uterine weight at peak infection although embryo number and viability are unchanged

Although Mal+ dams produced live pups at term, non-aborting Mal+ mice deviated from the normal pattern of gestational weight gain between GD/ED 9 and GD/ED 11. As this corresponds with the achievement of peak parasite burdens in Mal+ dams, animals were euthanized at GD 10 to interrogate the impact of maternal infection on conceptuses at this timepoint.

Parasite burden, hematocrit, and weight change between GD/ED 0 and GD/ED 10 were similar for cohorts of mice sacrificed at GD/ED 10 and GD/ED 18 (Supp. Fig. 5). Infection was not associated with midgestational abortion in gravid Mal+ dams at or prior to GD/ED 10. All successfully mated Mal+ and Mal− mice that exhibited pregnancy-associated increase in body weight ≥10% by GD/ED 8 were pregnant at sacrifice.

**Figure 5:**
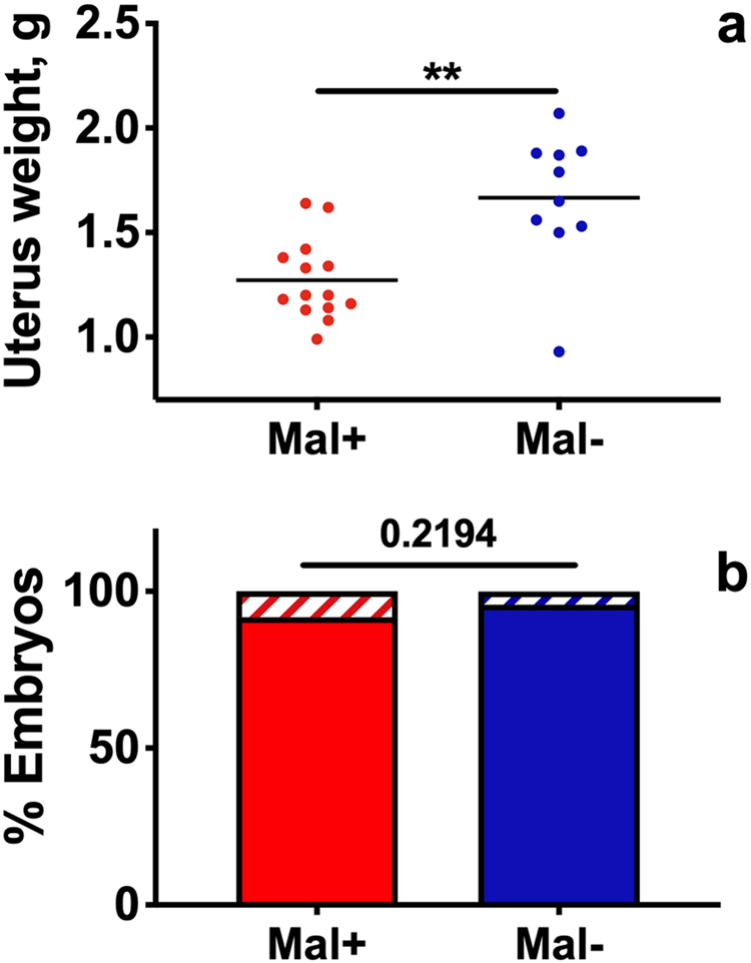
Embryo viability and uterine weight at GD/ED 10. Mal+ and Mal− dams were sacrificed at GD 10 for assessment of intrauterine development. (**a**) Uterine weights are presented pooled by dam infection group. Infection is associated with a significant reduction in uterine weight at GD/ED 10 (*P* < 0.01; Mann-Whitney U test). **P ≤ 0.01. (**b**) The proportion of viable (solid bars) and nonviable (hatched bars) embryos are presented as proportions of total embryos produced by the populations of Mal+ and Mal− dams. Infection is not associated with a significant reduction in embryo viability at GD/ED 10 (*P* = 0.2194; two-tailed Fisher exact test). Gravid Mal+ *n* = 157 fetuses and placentae, 14 litters; Gravid Mal− *n* = 119 fetuses and placentae, 10 litters; ns *P* > 0.05.

To assess the extent to which intrauterine growth restriction contributed to the pattern of stasis in normal gestation weight gain observed in between GD/ED 9 and GD/ED 11, we weighed uteri collected from Mal− and Mal+ dams sacrificed at GD/ED 10. This assessment showed that uteri derived from Mal+ dams weighed significantly less than uteri collected from Mal− controls (Fig. 5a). Notably, the reduction in uterine weights observed in Mal+ dams was not conclusively explained by embryo loss, as dams in both groups produced similar numbers of total embryos on average (Mal− dams (*n* = 10), 12 ± 2 embryos (mean ± SD); Mal+ dams (*n* = 14), 11 ± 1 embryos; *P* = 0.2956, Student’s *t* test). Likewise, Mal− dams produced 12 ± 2 viable embryos, while Mal+ dams produced 10 ± 2 (*P* = 0.0669, Mann-Whitney U test). Similarly, at the population level, malaria infection severity did not influence embryo viability at GD/ED 10 (Fig. 5b). A cohort of 14 Mal+ dams produced 157 embryos, 144 of which were viable (92%), while 10 Mal− dams produced 119 embryos, 114 of which were viable (96%; *P* = 0.2194; two-tailed Fisher exact test).

### Placentae of *P*. *chabaudi chabaudi* AS-infected Swiss Webster mice exhibit pathological features associated with human gestational malaria

The accumulation of iRBCs in the maternal blood spaces and the deposition of hemozoin in the placenta are hallmarks of gestational malaria in humans and are observed in some mouse models for malaria infection in pregnancy^34,41,42^. To determine if iRBCs and hemozoin were accumulating in the placentae of Mal+ dams around the time of peak infection (at GD 10), when stasis in weight gain was observed, mice were sacrificed and placentae processed for histological assessment.

To estimate placental parasite burden, iRBCs were counted in maternal blood sinusoids of placentae using Giemsa-stained tissue sections. Within Mal+ dams, though placental parasite burdens were higher than peripheral parasite burdens in 7/11 mice, the parasitemias in the two sites were not significantly different overall (Supp. Fig. 6). Manual counting of parasites in placental sections is not a highly accurate method of determining placental parasite burden for a number of technical reasons. Among these, in 7-micron tissue sections, sectioned iRBCs may not have detectable parasite material within the portion of the cell that is captured in the slide and therefore would not be counted as infected. Furthermore, *P*. *chabaudi chabaudi* AS parasites are synchronous, and dams in this study were not sacrificed at a time of day when a large proportion of the parasites would be at the relatively mature stages associated with tissue sequestration^39,40^. However, these data demonstrate that high parasite burdens are detectable in the placenta at GD/ED 10, indicating that placental infection is present at midgestation in this experimental model.

**Figure 6:**
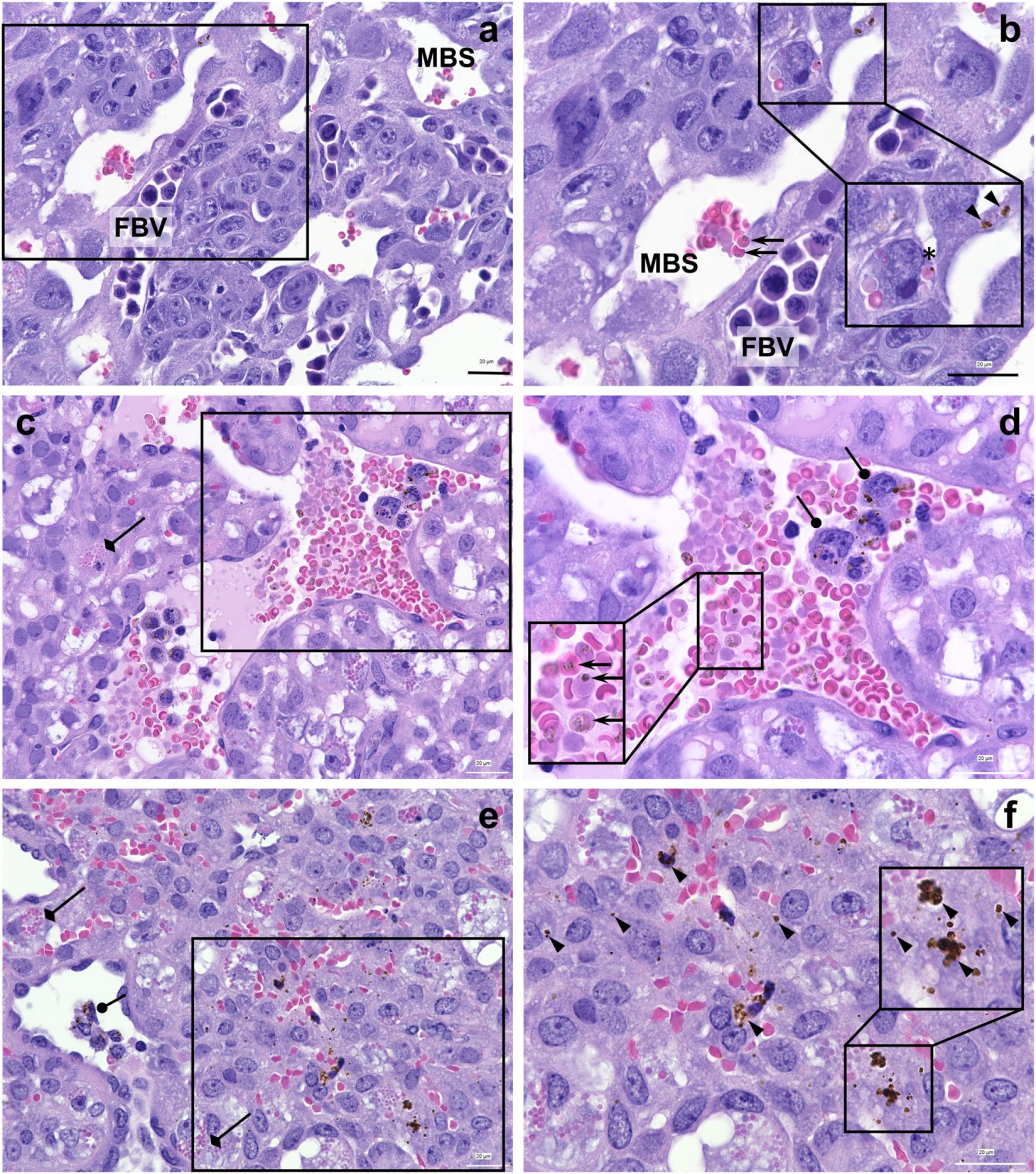
Micrographs of placentae at GD/ED 10. (**a**–**d**) Placental labyrinth region (**a**,**b**) and junctional zone (**c**,**d**) of a dam with 23.0% peripheral and 31.6% estimated placental parasitemia at GD 10. (**e**,**f**) Junctional zone of a dam with 14.4% peripheral and 20.2% estimated placental parasitemia at GD 10. Arrows depict examples of iRBCs in maternal blood sinusoids; examples of hemozoin in trophoblast is denoted by arrowheads and in maternal inflammatory cells by blunt arrows. Trophoblastic erythrophagocytosis is also evident (examples depicted with diamond arrows), including a whole iRBC (panel (b), asterisk). Demarcated areas in panels (a, c and e) (captured with a 60X objective) are represented as higher power images (captured with a 100X objective) in panels (b, d and f); bar = 20 μm. Insets in b, d and f are sized as indicated. FBV = fetal blood vessel; MBS = maternal blood sinusoid.

To measure hemozoin deposition in malaria-infected placentae, micrographs of placentae collected from Mal+ and Mal− dams underwent image analysis to quantify hemozoin. Consistent with the high parasite burdens observed in the placentae and periphery of Mal+ dams at GD/ED 10, abundant hemozoin was observed in Mal+ placentae (Table 1). By our image analysis technique, a significant increase in the area occupied by hemozoin was observed in Mal+ placentae compared to the background observed in Mal− placentae (*P* = 0.0167; two-tailed Student’s *t* test). Intracellular hemozoin was present within iRBCs in maternal blood sinusoids (Fig. 6a–d) and trophoblast (Fig. 6) in both the labyrinth (Fig. 6a,b) and junctional zones (Fig. 6c–f) of the placenta, and in maternal phagocytic cells (Fig. 6c,d). The presence of hemozoin within trophoblast is consistent with the erythrophagocytosis of *P*. *chabaudi*-iRBCs, which has previously been reported *in vivo* and in culture^41,55^.

**Table 1:** Placental hemozoin quantification at GD/ED 10.

Mouse #	Malaria status	Total # of MBV	Total # of images	% images with MBV	Total Hz area detected (mm^2^)	Total area assessed (mm^2^)	% area with Hz	Group mean ± SD	*P*
21452	Mal+	10	102	9.8	664	3528372	0.01882	0.04327 ± 0.02655	0.0167
21964	Mal+	12	101	11.9	1158	3707502	0.03123		
22242	Mal+	16	88	18.2	978	3073393	0.03182		
22238	Mal+	12	97	12.4	2867	3467525	0.08268		
22244	Mal+	11	98	11.2	2806	3668019	0.07650		
21451	Mal+	11	102	10.8	1551	3455204	0.04489		
21449	Mal+	10	93	10.8	598	3533365	0.01692		
21961	Mal−	8	25	32.0	60	911054	0.00659	0.00569 ± 0.00169	—
21956	Mal−	12	33	36.4	79	1173084	0.00673		
22235	Mal−	17	44	38.6	59	1576858	0.00374		

Placental hemozoin quantification data is presented for 7 Mal+ and 3 Mal− dams. The percent area occupied by hemozoin was compared between Mal+ and Mal− dams by two-tailed Mann-Whitney U test. MBV = maternal blood vessel; Hz = hemozoin.

Image analysis of the placental junctional zone also revealed that malaria infection was associated with a significant decrease in the percentage of micrographs containing maternal blood vessels (Table 1), as has previously been reported in *P*. *chabaudi chabaudi* AS and *P*. *berghei* infected mice^34,56^. Among Mal+ dams, 9.8% to 18.2% of placental micrographs contained maternal blood vessels, while maternal blood vessels were observed in 32.0% to 38.6% of placental micrographs of Mal− placentae (*P* < 0.0001; two-tailed Mann Whitney U test).

### Transcription of an oxidative stress gene is increased in malaria-exposed conceptuses

Gestational malaria is associated with placental oxidative stress in both humans and mice^22,54,57^. To measure placental antioxidant responses in this model, conceptuses collected from Mal+ and Mal− dams at GD/ED 10 were evaluated by quantitative reverse transcription PCR (qPCR). Transcripts of the antioxidant genes *Sod1*, *Sod2*, *Sod3*, and *Cat* were analyzed. These genes encode superoxide dismutases (1–3) and catalase, all of which interact with and neutralize damaging radicals. The transcription of *Nrf2*, the gene encoding nuclear factor (erythroid-derived 2)-like 2, a transcription factor involved in the response to oxidative stress, was also measured. Finally, the gene encoding heme oxygenase, *Hmox1*, the enzyme that catalyzes the degradation of toxic free heme, was assessed. *Hmox1* is especially significant in the context of malaria infection because the destruction of iRBCs releases free heme, a potent oxidant, into circulation.

**Figure 7:**
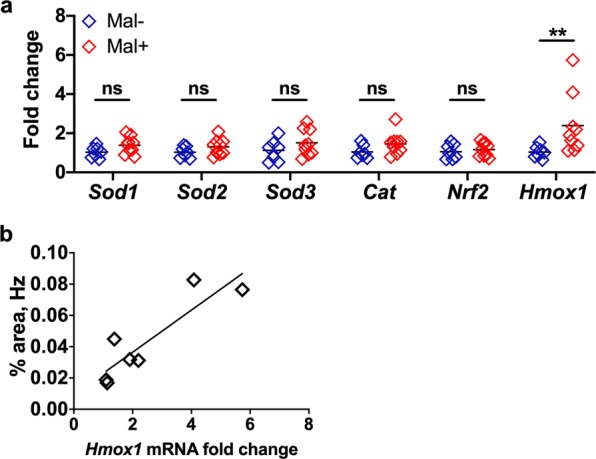
Malaria-induced expression of antioxidant response genes in *P*. *chabaudi chabaudi* AS-exposed conceptuses. (**a**) Transcript levels of mouse *Sod1*, *Sod2*, *Sod3*, *Nrf2*, and *Hmox1* were quantified by qPCR. mRNA abundance was internally normalized to *Ubc* expression and is presented relative to expression in control conceptuses that were not exposed to malaria infection. Group means and transcript abundance in individual mice are depicted. Infection was associated with increased transcript abundance of *Hmox1* (*P* ≤ 0.01; Mann-Whitney U test). Infection does not alter the abundance of *Sod1*, *Sod2*, *Sod3*, or *Nrf2* transcripts (*P* > 0.05; Mann-Whitney U test). Gravid Mal− *n* = 7; Gravid Mal+ *n* = 9, ** *P* ≤ 0.01. (**b**) The transcript abundance of *Hmox1* in conceptuses versus placental hemozoin density is depicted for a subset of Mal+ dams sacrificed at GD/ED 10. Hemozoin density was determined by image analysis and is presented as the percentage hemozoin-occupied area in micrographs of the junctional zone. A significant positive relationship was detected between *Hmox*1 expression and hemozoin density by linear regression analysis (*R*^*2*^ = 0.7908, *P* = 0.0074). Gravid Mal+ *n* = 7.

The expression of *Sod1*, *Sod2*, *Sod3*, *Cat*, and *Nrf2* did not differ significantly between conceptuses collected from Mal+ dams and conceptuses collected from Mal− controls (Fig. 7a). The expression of *Hmox1* was significantly increased in Mal+ dams compared to Mal− controls (Fig. 7a), perhaps reflecting a compensatory mechanism seeking to neutralize free heme and hemozoin released by the rupture of iRBCs. Consistent with this hypothesis, the expression of *Hmox1* is strongly associated with hemozoin density as determined by image analysis (Fig. 7b).

## Discussion

Mouse models of malaria infection rely heavily on inbred mice, although different strains can vary dramatically in the response to a given infection as a result of their innate immunological biases^58,59^. Restricting studies of malaria to inbred mice therefore limits the ability of scientists to identify and characterize responses to infection that transcend genetically diverse backgrounds that are represented in outbred human populations. Outbred mice are a potential option to overcome this limitation but have not been extensively used for the study of malaria or other infectious diseases for a number of reasons. First, the genetic variability of outbred mouse stocks has not been rigorously explored^60^, leading to concerns about genetic quality control. Second, the inherent diversity of an outbred mouse stock might yield a more diverse range of outcomes, necessitating the use of larger cohorts of animals and increasing experimental costs. Finally, outbred mice are not appropriate for the study of some genetically determined traits, as phenotypic variability may reflect genetic diversity, not experimental treatment. Nonetheless, outbred mice by their very nature should faithfully represent the breadth of disease responses within genetically diverse populations. It follows that responses shared by many of the members of the population may be illuminating, because they may represent universally relevant mechanisms. This was a major motivation for using Swiss Webster mice to develop a novel model for understanding critical elements of malaria pathogenesis in pregnancy that transcend host genetic variability. As reported here, this effort has revealed these mice to be an important addition to already available model systems based on inbred mice to explore the impact of malaria in pregnancy.

Animal models for gestational malaria have been vital to advancing understanding of how maternal malaria infection impacts pregnancy success^61^, studies of which can be challenging in human populations. In human malaria, infants exposed to malaria infection *in utero* face an elevated risk of death due to fetal growth restriction, preterm delivery, and low birth weight^1–6,48^. These poor birth outcomes are attributed to placental dysfunction stemming from the damage sustained by the placenta during malaria infection. At delivery, the accumulation of iRBCs and maternal immune cells^23–25^, hypercoagulation^46,62^, syncytial knotting and rupture^23,24,26^, and villous tissue necrosis^23–26^ are observed in placenta impacted by *P*. *falciparum*, suggesting that placental damage due to malaria is profound even when the pregnancy culminates in a live birth. Given the difficulties inherent in gaining a mechanistic understanding of pathogenic processes at the placental level in human malaria, and how these processes lead to poor birth outcomes, mouse models are an essential tool. The most widely used models entail infection of a naïve mouse resulting in abortion, preterm delivery, or stillbirth^34,36–38,41–46^. As the impact of exposure to maternal malaria infection *in utero* on child development is one of the primary public health concerns associated with gestational malaria, the inability of these models to produce live progeny is a major limitation. The existing models for generating malaria-experienced pups utilize infection with *Plasmodium berghei* or heterologous *P*. *chabaudi* infection. When *P*. *berghei* is used, dams must be infected after midgestation to allow the fetuses to reach gestational term prior to pregnancy loss or maternal death^34,38,63^. Progeny exposed to *P*. *berghei* during gestation must be fostered due to maternal mortality^34,63^. In heterologous infection with nonlethal *P*. *chabaudi chabaudi* strains CB and AS, infection of B6 dams with the former prior to pregnancy, followed by infection with the latter on GD 10 allows the mice to proceed to term and deliver live pups following the development of significant parasite burdens^49^. An important strength of this heterologous infection model is that it allows for the exploration of gestational malaria in a malaria-experienced dam, better modeling gestational malaria in a malaria-endemic region. Moreover, the mice develop relatively low parasite burdens more homologous to those observed in *P*. *falciparum*-infected humans. However, this model has only been described in inbred mice (B6) and requires monitoring infected dams throughout two infections, resulting in a protracted experimental timeline^49^. In addition, in both models utilizing heterologous *P*. *chabaudi* or *P*. *berghei* infection, significant parasite burdens are observed only in the last third of the pregnancy, not throughout gestation^34,49,63^. An important feature of the model reported herein is a peak of susceptibility at midgestation, which was reported in early studies of malaria during human pregnancy in Africa^64^. Additionally, mice remain infected throughout gestation, which is characteristic of human infection in malaria endemic areas^65,66^. It is important to note that the Swiss Webster mice used in this model, unlike women living in malaria-endemic regions, have no previous exposure to malaria infection. The extent to which prior exposure will influence subsequent infections in the context of pregnancy is an important next step for development of this model.

Following infection with *P*. *chabaudi chabaudi* AS on GD/ED 0, gravid Swiss Webster mice developed high parasite burdens and severe malarial anaemia. Around the time of peak infection (GD/ED 10), high parasite burdens were observed in Mal+ placentae but estimated placental parasitemia did not universally exceed that observed in the periphery. Historically, the accumulation of iRBCs in the placenta has been associated with gestational malaria in both humans and mice. In humans, placental accumulation of *P*. *falciparum*-iRBCs is linked to placental pathology and poor birth outcomes^20,21,23,24^. Alternatively, infection with *P*. *vivax* is not typically associated with placental sequestration, although the binding of *P*. *vivax*-iRBCs to placental tissue has been reported *in vitro*^18^. Despite a dearth of strong evidence for placental accumulation of iRBCs, *P*. *vivax* infection during pregnancy has also been associated with placental pathology and poor birth outcomes^3,17,67^. Thus, placental sequestration of iRBCs may not be absolutely required to observe placental dysfunction and poor birth outcomes in malaria-infected women. Murine-infective *P*. *berghei* and *P*. *chabaudi* are also cytoadherent and accumulate in the placentae of infected dams^34,41,55,68^. In *P*. *berghei*-infected dams, iRBC binding within the placenta is mediated by the expression of CSA and hyaluronic acid on the trophoblast as observed in *P*. *falciparum*-infected women^34^. Definitive cytoadherence and relevant placental ligands for *P*. *chabaudi chabaudi* AS-iRBCs remain to be characterized. In the presently described model, further study is required to determine the kinetics and mechanisms of parasite accumulation in the placenta, though we posit that placental sequestration may not be necessary to drive placental pathology in a malaria-infected dam.

Consistent with the high parasite burdens observed in the periphery and the maternal blood spaces of the placenta, significant hemozoin deposition was observed in Mal+ placentae at GD/ED 10, associated with the elevated expression of *Hmox1*. Upregulation of this gene is considered a biomarker for oxidative stress and is proposed to be essential for optimal placental function^69^. Placental oxidative stress, and the lipid peroxidation that results, has previously been reported in both human and murine gestational malaria^22,54,57^. Moreover, elevated heme and heme oxygenase 1, exacerbated by iron supplementation, was reported in malaria-infected pregnant women, with heme being higher in women who delivered preterm^70^. In the studies described here, we observe a correlation between placental hemozoin density and *Hmox1* transcript abundance in malaria-exposed conceptuses. Hemozoin, like free heme, is released by the rupture of iRBCs. The observed relationship between hemozoin density and *Hmox1* transcript abundance may reflect the combined release of both forms of iron in the placenta. However, in a murine model for malaria-associated acute respiratory distress syndrome, the intravenous injection of hemozoin into uninfected mice was associated with an increase in the expression *Hmox1* in the lungs^71^, suggesting that hemozoin itself promotes oxidative stress. The precise relationships between heme, hemozoin, and oxidative stress in the context of malaria in pregnancy is an interesting phenomenon that requires further study.

Although most Mal+ dams carried their pregnancies to term and produced live progeny, some failed to gain weight after midgestation, around the time of peak infection (GD/ED 10 to GD/ED 11), and produced no progeny; it is concluded that these were abortions. The precise timing of pregnancy loss in these animals is unclear, as the majority of these animals were not sacrificed at midgestation. The uteri of these dams at term (GD/ED 18) did not contain resorptions, indicating that the embryos were likely expelled earlier in gestation. Among those that were sacrificed at GD/ED 10, none exhibited signs of abortion and embryo expulsion. Neither vaginal discharge nor bloody bedding were observed at any time during infection, so definitive attribution of the observed weight loss at midgestation to spontaneous abortion and the expulsion of embryonic tissue is not possible at this time. The extent to which inflammation and oxidative stress associated with malaria infection contributes to pregnancy loss in these animals, as previously observed in other mouse models for gestational malaria^54,57^ remains to be demonstrated, but upregulated expression of *Hmox1* in mice examined at midgestation is consistent with this conclusion. Moreover, in a related study, we recently found that severity of malaria and pregnancy outcomes can be modulated by altering the gut microbial communities in Swiss Webster mice. In this work, severe infection was associated with significant hemozoin accumulation, elevated transcripts for *Ifng*, *Il10*, and *Mgl2*, a marker for monocyte/macrophages in conceptuses, and reduced maternal vascular space in the placenta. These findings were coincident with reduced fetal weight at term and reduced survival in the first four days of life^56^. In addition to placental pathology, a stasis in pregnancy-associated weight gain was observed in infected mice between GD/ED 9 and GD/ED 11. Remarkably, most remained pregnant despite developing parasite burdens exceeding those observed in *P*. *chabaudi chabaudi* AS-infected B6 mice that abort their pregnancies^41–45^. Uterine weights at GD/ED 10 were reduced by infection, but embryo number and viability were not significantly altered. Mal+ dams ultimately recovered and produced live progeny, but fetal weight at term (GD 18) was reduced in infected dams. The reduction in midgestational uterine weight and GD/ED 18 fetal weights in Mal+ dams suggests that intrauterine growth restriction contributes to the reduction in gestational weight gain observed in gravid Mal+ mice. However, the potential contributions of anorexia, malaria-related metabolic disruption, or other maternal behaviors and physiological processes cannot be excluded. A recent study describing the infection of GD 12 Swiss Webster mice with *P*. *berghei* ANKA^GFP^ provides evidence that both maternal and fetal factors contribute to infection-related deficits in gestational weight gain, as both reduced fetal body weights and reduced maternal body weights (excluding the weight of the uterus) were observed in infected mice^38^. These parameters remain to be understood in human infections as well; further work in this and other mouse models will continue to be important in advancing understanding of these contributors to malaria pathogenesis in pregnancy.

Human gestational malaria has been associated with preterm delivery; however, the term delivery of infants that are underweight due to intrauterine growth restriction is observed more frequently^3,72^. Similarly, we did not consistently observe preterm delivery due to *P*. *chabaudi chabaudi* AS infection. Interestingly, Mal+ dams delivered their litters between GD 18 and GD 21, whereas Mal− dams all delivered on GD 19, but the gestation durations of the two groups were not statistically significantly different. It is notable, however, that the two dams in the Mal+ cohort that produced litters that did not survive until 4 days of age spontaneously delivered on GD 18 and GD 19 following loss of weight, while all Mal+ mice that delivered pups that survived continued to gain weight and delivered on GD 19 or later. This observation may suggest that preterm delivery occurs at low frequency in this model and may be associated with reduced neonatal survival. Further study is required to dissect the relationship between maternal malaria infection and the onset of delivery, and the extent to which genetic loci that vary in these mice are an important determinant.

While the average number of viable fetuses found in Swiss Webster dams at GD 18 was not reduced by *P*. *chabaudi chabaudi* AS infection, the average number of pups present at weaning was significantly reduced in the litters of Mal+ dams. In this model, the majority of pup deaths occurred between birth and 4 days of age. Pup mortality is not universally observed in other models for the production of malaria-exposed progeny. Infection with *P*. *berghei* ANKA was associated with increased pup mortality between 2 and 21 days of age, although the timing of pup deaths was not described^34^. In contrast, heterologous infection with *P*. *chabaudi* during pregnancy was not associated with increased pup mortality between birth and weaning. The variable pup survival outcomes observed in these models may again reflect differences in experimental design, or they may reflect inherent differences between parasite species and mouse strains or stocks. Infection in the models described above were initiated on GD 10 and GD 13, respectively, whereas infection is initiated on GD 0 in the novel model described here. The timing of infection initiation and peak parasite burden may contribute to postnatal growth phenotypes. Alternatively, these differences may be attributable to innate differences between inbred BALB/c or B6 mice and outbred Swiss Webster mice or to innate differences between strains CB and AS of *P*. *chabaudi chabaudi* AS or *P*. *berghei*.

There are three likely explanations for the contraction in Mal+ litter size we observe in the perinatal period between GD 18 and 4 days of age. First, fetal viability at GD 18, as determined by reactive movement, may be a poor predictor of postnatal survival, and despite live birth, these neonates may be less fit due to *in utero* malaria exposure. Second, Mal+ dams may exhibit reduced nourishing capacity due to their infection, resulting in an increased rate of neonatal pup loss. Relatedly, Mal+ dams may be more likely to cannibalize their young or fail to care for them appropriately in the first days of life. Although we did not weigh pups at delivery, we speculate that intrauterine growth restriction observed in GD/ED 18 fetuses produced by Mal+ dams results in the birth of underweight pups, and that low birth weight is associated with an elevated risk of neonatal death. A related study that we conducted recently supports this idea, and suggests that this impact likely outweighs maternal post-partum behavior. Neonates born to Swiss Webster dams that exhibited patterns of infection like those reported here displayed the same tendency to perish during the first four days of life, even when fostered on uninfected Swiss Webster dams^56^. In humans, low birth weight is associated with a significantly higher risk of infant death, regardless of etiology. In the early 2000s, low birth weight was estimated to account for 60–80% of neonatal deaths, although low birth weight was only observed in approximately 14% of births globally^73^. The highest rates of neonatal mortality were observed in Sub-Saharan Africa^73^, likely reflecting the burden of *P*. *falciparum* infection in the region. Maternal malaria infection is a major cause of low birth weight, and therefore, a major cause of infant death in malaria-endemic regions^2–5^. Notably, a large proportion of these deaths occur within the neonatal period, or the first month of life^2,3^. Consequently, the novel mouse model described here may recapitulate this outcome of gestational malaria infection: increased risk of death in early life due to restricted fetal growth. However, it is also possible that maternal malaria infection results in other developmental delays in malaria-exposed fetuses resulting in an increased risk of death due to inadequate prenatal development. Given the interest in elucidating the impact of *in utero* malaria exposure on infant outcomes, such as neurological development^10,63^, this model presents an exciting opportunity to identify universal determinants in both mother and offspring that transcend a variable genetic background to produce poor outcomes.

Despite the attrition in pup number observed in the litters of Mal+ dams prior to 4 days of age, the surviving progeny gained weight normally between birth and weaning. The number of pups in the litter at weaning and the starting weight of the dam significantly influence the trajectory of pup growth, but neither maternal infection status nor pup sex has a significant impact. This is inconsistent with the previously described model for heterologous *P*. *chabaudi* infection during gestation, where heterologous infection during pregnancy was associated with reduced pre- and post-weaning weights in progeny^49^. Similarly, infection with *P*. *berghei* ANKA in midgestation reduces pre-weaning growth in the progeny of Mal+ BALB/c dams^34^. The variable impact of maternal infection on pup growth observed in these models may again reflect differences in experimental design, or they may reflect inherent differences between parasite species and mouse strains or stocks.

The underlying mechanisms that allow gravid Swiss Webster mice infected with *P*. *chabaudi chabaudi* AS in early gestation to carry pregnancies to term remain to be explored. We anticipate that future studies will allow for the elucidation of host responses that control infection and preserve pregnancy success and those that contribute to fetal loss during maternal infection. In addition, future studies probing the causes of neonatal death in malaria-exposed progeny may enhance our understanding of the impact of maternal malaria infection on infant survival. In the context of human infections, we believe that the study of outbred mouse models is important, as commonly observed responses may be universal phenomena, not artifacts observed in a murine population as a result of inbreeding. These future mechanistic studies, as well as the further characterization of the impact of transgestational maternal malaria infection on fetal and postnatal outcomes, is expected to advance our understanding of placental dysfunction during gestational malaria.

## Materials and Methods

### Mice

Seven- to ten-week-old female Swiss Webster mice and eight- to ten-week-old male Swiss Webster mice (Clr:CFW) were purchased from the National Cancer Institute Mouse Repository (Frederick, MD). Mice were maintained under specific-pathogen-free conditions, in *Helicobacter* species and norovirus-free rooms, at the University of Georgia Coverdell Vivarium, a barrier facility. Husbandry and experiments were approved by and performed in accordance with guidelines and regulations set forth by the University of Georgia Institutional Animal Care and Use Committee. All animals were supplied food (PicoLab Mouse Diet 20 5053; St. Louis, MO) and water ad libitum. Mice were adjusted to a 14-hour light/10-hour dark cycle and housed in conditions of 65–75 °F and 40–60% humidity.

### Parasites and infection monitoring

The following reagent was obtained through BEI Resources Repository, NIAID, NIH: *Plasmodium chabaudi chabaudi*, Strain AS, MRA-741, contributed by David Walliker. Parasites were maintained as frozen stock in accordance with supplier guidelines and amplified in A/J mice for the purposes of infecting experimental Swiss Webster mice.

Peripheral parasitemia was estimated by flow cytometry using a method adapted from Jimenez-Diaz *et al*.^74^. Blood samples were collected by tail-clip^75^, diluted 1:50 in isotonic saline, and stained within 4 hours of collection with 2.5 μM SYTO® 16 Green Florescent Nucleic Acid Stain (ThermoFisher Scientific; Waltham, MA). Samples were incubated at room temperature in the dark for 20 minutes, diluted 1:9 in isotonic saline, and analyzed using a CyAn ADP Flow Cytometer (Beckman Coulter; Brea, CA) within 4.5 hours of collection. Thirty thousand cells were analyzed per mouse, and iRBCs were identified by size and fluorescence intensity. A stained sample of uninfected blood was analyzed alongside infected blood samples on each day of measurement to serve as an internal control. Parasitemia is reported as the percentage of iRBCs in the total number of counted red blood cells.

### Experimental design

Eight- to ten-week-old female Swiss Webster mice were paired with sexually mature Swiss Webster males. Each morning, female mice were examined for the presence of a vaginal plug, indicating successful mating. The day a vaginal plug was observed was considered GD 0. Successfully mated female mice were distributed randomly into Mal+ and uninfected Mal− cohorts. Age-matched mice that had not been mated were infected as virgin controls. Body weight and hematocrit were measured immediately prior to infection. Gravid and virgin Mal+ mice were infected intravenously with 10^3^ *P*. *chabaudi chabaudi* AS-iRBCs in 200 μl 1x phosphate-buffered saline (PBS) per 20 grams of body weight. Gravid Mal− control mice were intravenously injected with 200 μl 1x PBS per 20 grams of body weight to control for stress and handling. At the time of infection or mock infection, all experimental animals, regardless of pregnancy status, were transitioned to a higher fat chow (PicoLab Mouse Diet 20 5058; St. Louis, MO) better suited to the nutritional needs of a pregnant or lactating mouse. Following the measurement of weight and hematocrit immediately prior to infection at GD/ED 0, mice were left undisturbed until GD/ED 6 to maximize blastocyst implantation (GD 4.5) and pregnancy success. Beginning at GD/ED 6, body weight, hematocrit, and parasitemia were measured daily until GD/ED 10 or GD/ED 18 to assess the development of infection and pregnancy progress. Mated female mice displaying a 10% or greater increase in body weight between GD 0 and GD 8 were considered gravid.

Mice were anesthetized with 2.5% Tribromoethanol prior to sacrifice at midgestation, GD/ED 10, or gestational term, GD/ED 18. In dams sacrificed at GD/ED 10, plump, pink, well-vascularized embryos without hemorrhaging were considered viable. Nonviable embryos and resorptions were included in the count of total embryos. Approximately half of the conceptuses were removed from the uterus and snap frozen for gene expression analysis, while the remaining conceptuses were preserved in formalin within the uterus for histology. In dams sacrificed at GD/ED 18, fetal viability was determined by the assessment of reactive movement in response to prodding with blunt-tipped forceps. Viable and nonviable fetuses were counted, and viable fetuses and their placentae were individually weighed.

In an alternative study design, gravid Mal+ and Mal− mice were allowed to proceed to spontaneous delivery of the litter. Dams were single housed beginning at GD 17 and cages were checked at least twice daily for pups beginning at GD 18. Pups were not counted until 4 days of age because maternal distress within the first few days of life can result in the rejection of the litter. At four days of age, pups were uniquely identified by footpad tattooing with nontoxic carbon pigment ink (Super Black Speedball India Ink; Statesville, NC)^76,77^. Individual pups were weighed at four days of age and every three days thereafter until weaning at 22 days of age. A single pup, produced by a Mal+ dam, perished between 11 to 13 days of age; due to incomplete growth trajectory data collected for this pup, its data were censored from postnatal growth analysis. Pup sex was visually determined at weaning.

### Placental parasitemia and hemozoin quantification

Uterine segments containing conceptuses were harvested at sacrifice and fixed overnight in 4% buffered formalin. Following overnight fixation, uterine segments were bisected longitudinally and allowed to fix for another 24 hours to complete fixation of tissues. Tissues were then dehydrated with ethanol, cleared in xylenes, and infiltrated in melted paraffin prior to embedding. Seven micron sections were Giemsa-stained, and parasite burden in the placenta was measured by counting iRBCs in junctional zone maternal blood sinusoids in Giemsa-stained tissue sections. At least 10^3^ erythrocytes were counted for each dam across a minimum of three conceptuses. Placental parasitemia is presented as the percentage of iRBCs counted in the maternal blood. Alternatively, 7 micron tissue sections were stained with hematoxylin and eosin for the assessment of histopathological features, including hemozoin deposition and Giemsa for optimal visualization of iRBCs. Micrographs were captured using Nikon Eclipse E400 microscope with a Keyence BZ-X710 microscope using 60x or 100x objectives as indicated.

Giemsa-stained tissue sections were further assessed for hemozoin deposition in the placental junctional zone using a Keyence BZ-X710 microscope with BZ-X Analyzer software. Eighty-eight to one hundred two 242 μm x 181 μm areas were captured across 3–5 individual Mal+ placentae using a 60X oil objective. Among these images, 9.8–18.2% (mean ± SD: 12.2 ± 2.8%) included maternal blood sinusoids containing hemozoin-bearing infected RBCs and maternal inflammatory cells. To validate this technique and demonstrate the low incidence of pixels detected as hemozoin in Mal− mice, twenty-five to forty-four 242 μm × 181μm areas were also captured across 2 individual Mal− placentae. Representative images from one of these Mal− placentae is provided in Supp. Fig. 7. BZ-X Analyzer software was used to exclude acellular spaces, and, based on hue, area occupied by hemozoin within cells (RBCs, maternal inflammatory cells, trophoblast) and in fibrin was estimated. Hemozoin-occupied area is presented as a percent of total evaluated space, which measured overall from 3,073,393 to 3,707,502 μm^2^ for each assessed series of Mal+ embryos, and 911,054 to 1,576,858 μm^2^ for each assessed series of Mal− embryos. Hemozoin scoring data is presented in Table 1.

### Gene expression

Total RNA was isolated from GD/ED 10 mouse conceptuses using the RNeasy Plus Mini Kit (Qiagen; Germantown, MD) following homogenization in lysis buffer using the TissueMiser (Fisher Scientific; Waltham, MA) or Bullet Blender Gold (Next Advance; Troy, NY). A minimum of 3 conceptuses were pooled per dam. Reverse transcription was performed using the High-Capacity cDNA Reverse Transcription Kit (Applied Biosystems; Foster City, CA). Relative transcript abundance was measured using Power SYBR® Green Supermix (Applied Biosystems; Foster City, CA) with the C1000 Touch Thermal Cycler (CFX96 Real Time Systems; Bio-Rad, Hercules, CA). Each sample was assayed in duplicate for all target and housekeeping genes. Average threshold cycle (Ct) values were normalized to average *Ubc*^78,79^ Ct values. Relative transcript abundance of each gene of interest was determined using the ΔΔCt method^80^. Briefly, transcript expression in individual mice is presented relative to the mean expression value in Mal− mice. When possible, primer pairs were designed to span large introns such that amplification exclusively represented cDNA template. Primer sequences and amplicon sizes are listed in Supplemental Table 1.

### Statistics

Descriptive statistical analyses were performed using GraphPad Prism 7 (GraphPad Software; La Jolla, California). All raw clinical data are presented as mean ± SEM. Error bars are not depicted if the error bars are shorter than the height of the symbol. The area under the curve (AUC) of percent starting weight, hematocrit, and parasitemia was calculated for each mouse between GD 0 and GD 18 and is presented as a scatter plot with a bar representing the mean. Parametric statistical tests were utilized only if the data were determined to be normally distributed using the D’Agostino-Pearson normality test. Parasitemia AUC values between gravid Mal+ and virgin Mal+ cohorts were compared using a two-tailed Student’s *t* test. Percent starting weight and hematocrit AUC values were analyzed by one-way analysis of variance (ANOVA) followed by Bonferroni’s multiple comparison test between gravid Mal− and gravid Mal+ cohorts and gravid Mal+ and virgin Mal+ cohorts. Starting weight AUC values for Mal+ gravid and aborting Mal+ mice were compared by Mann-Whitney U test. The number of total and viable fetuses produced by Mal+ and Mal− dams were compared using a two-tailed Student’s *t* test. Fetal and placental weights, pooled by dam infection status, are presented as box and whisker plots with whiskers extending from the 10^th^ to the 90^th^ percentile for visualization only. Outliers are depicted as individual data points. Gestational duration of Mal+ and Mal− dams is presented as a Kaplan-Meier curve and groups were compared using the Mantel-Cox test. Fetal viability, embryo viability, and pup sex were compared across infection groups by two-tailed Fisher exact test. Uterine weights were compared by Mann-Whitney U test. Placental and peripheral parasitemia pairs were compared using Wilcoxon matched-pairs signed rank test. In micrographs collected to measure placental hemozoin deposition, the percent area occupied by hemozoin in Mal+ and Mal− placentae was compared by two-tailed Mann-Whitney U test, and the percent of placental images containing maternal blood vessels between Mal+ and Mal− placentae was compared by two-tailed Student’s *t* test. The relationship between *Hmox1* transcript abundance and hemozoin density was determined by linear regression analysis. The starting weights of mated mice assigned to Mal+ and Mal− cohorts were compared using a two-tailed Student’s *t* test. *P* values ≤ 0.05 were considered statistically significant.

Mixed linear models analysis (SAS 9.4) was used to estimate differences in term fetal and placental weights and postnatal pup growth as a function of dam infection. In both cases, the interrelatedness of pups born to the same dam was controlled with a random term for the dam. Best fit for the former model was obtained with inclusion of infection status (binary variable), dam starting weight (continuous variable) and number of viable pups in each dam (continuous variable). The latter model assessed trends over time for changes in pup growth; repeated measures in the pups were accommodated with a repeat command and the relatedness of pups born to the same dam was accounted for with a random term for the dam. Model fitting revealed that only dam weight at initiation of the experiment, number of pups at weaning, and age at weight measurement, with associated interaction terms with the latter, influenced trends in weight gain. Pup sex and maternal infection status were not significant in initial models but are depicted in the figure for illustration purposes.

## Supplementary information

Supplementary Information

Dataset 1

## Data Availability

All data generated or analyzed during this study are included in this published article and its Supplementary Dataset file.
