# Supplementary material for: A novel murine model for assessing fetal and birth outcomes following transgestational maternal malaria infection
Source: Sci Rep. 2019 Dec 20;9:19566. doi: 10.1038/s41598-019-55588-8 (PMC6925284; doi:10.1038/s41598-019-55588-8)
Supplement: Supplementary file 1 — Supplementary Information [file 41598_2019_55588_MOESM1_ESM.docx]

*A novel murine model for assessing fetal and birth outcomes following transgestational maternal malaria infection*

Catherine D. Morffy Smith^1^, Brittany N. Russ^2^, Alicer K. Andrew^3^, Caitlin A. Cooper^4^, Julie M. Moore^5,*^

1. Department of Infectious Diseases and Center for Tropical and Emerging Global Diseases, University of Georgia, Athens, GA; cdmorffysmith@gmail.com
2. Department of Infectious Diseases and Immunology, University of Florida, Gainesville, FL; bruss@ufl.edu
3. Department of Infectious Diseases and Center for Tropical and Emerging Global Diseases, University of Georgia, Athens, GA; andrewa5@uga.edu
4. Department of Infectious Diseases and Center for Tropical and Emerging Global Diseases, University of Georgia, Athens, GA; cooper13@uga.edu
5. Department of Infectious Diseases and Center for Tropical and Emerging Global Diseases, University of Georgia, Athens, GA; Current Institution: Department of Infectious Diseases and Immunology, University of Florida, Gainesville, FL; juliemoore@ufl.edu

* Corresponding author

**Supplemental data, Supplemental Figures 1-7; Supplemental Table 1.**

**Supplemental Figure 1. Gravid Swiss Webster mice display a 10% or greater increase in body weight at GD 8 regardless of infection status.**

Weight gain at GD 8 is presented for virgin Mal+, gravid Mal+, and gravid Mal- mice. Only gravid mice that produced live fetuses at term or delivered live pups are displayed. On the basis of the 10% minimum weight gain observed in these gravid mice, intended gravid mice were determined to be pregnant if they exhibited a 10% or greater increase in body weight at GD 8, regardless of the production of viable fetuses or live pups at term.

Virgin Mal+ *n* = 11; Gravid Mal+ *n* = 23; Gravid Mal- *n* = 18.

**
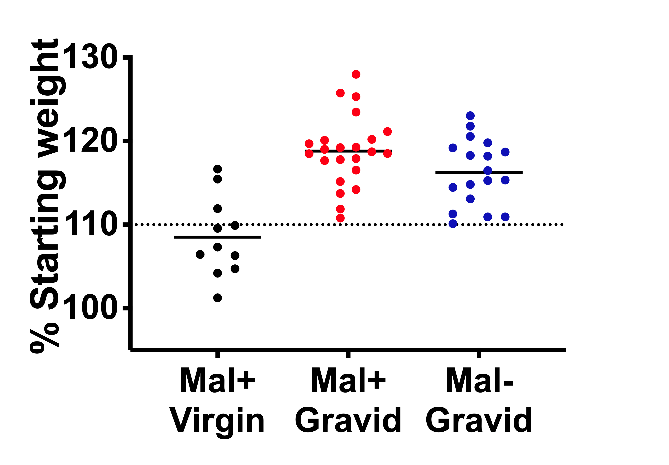
**

**Supplemental Figure 2. Fetal and placental weights by dam at GD 18.**

Numbers on the X-axis represent the unique identifier assigned to each dam.

- 1. Weights of viable fetuses per dam.
  2. Weights of placentae associated with viable fetuses per dam.

**
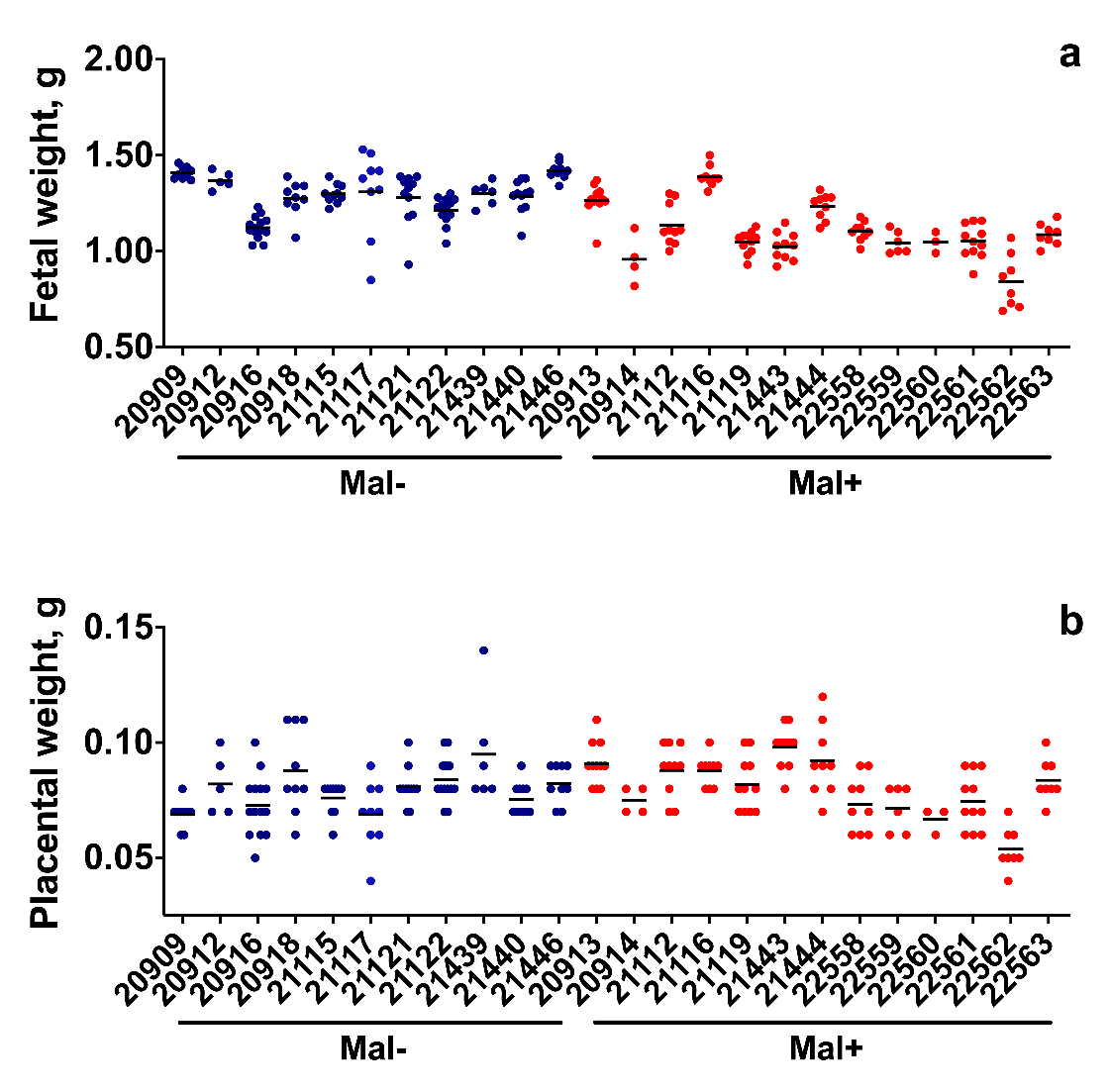
**

**Supplemental Figure 3. Dam weights at GD 0.**

No significant difference in mean body weight is observed between mice assigned to Mal+ and Mal- cohorts at the time of infection (*P* = 0.7296, two-tailed Student’s *t* test).

Gravid Mal+ *n* = 23; Gravid Mal- *n* = 18; ns *P* > 0.05.

**
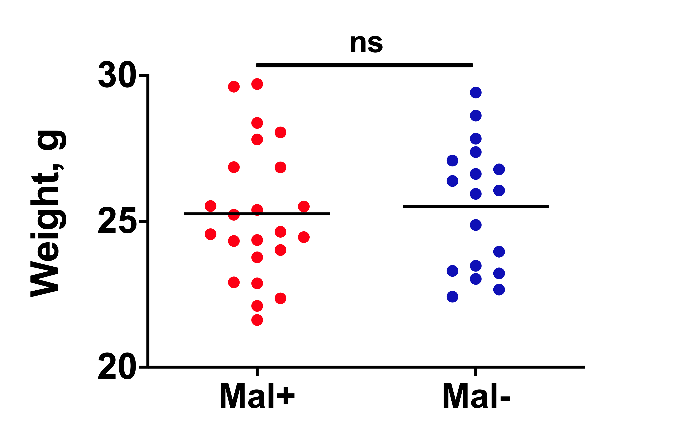
**

**Supplemental Figure 4. Relationship between litter size and average pup size at weaning.**

The number of pups in a litter versus the mean weight of a pup in a litter is presented for the litters of Mal+ and Mal- dams. A significant negative correlation is observed between pup number at weaning and the average weight of pups within the litter among Mal- litters (Pearson *r* = -0.8969, *P* = 0.0062). No significant correlation is observed within Mal+ litters (Pearson *r* = -0.493, *P* > 0.05).

Gravid Mal+ *n* = 9 litters; Gravid Mal- *n* = 7 litters.

**
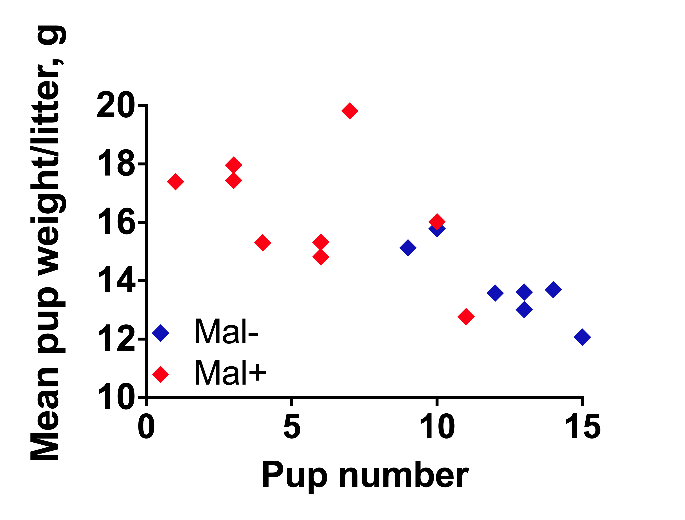
**

**Supplemental Figure 5. Parasitemia, hematocrit, and weight change in *P. chabaudi chabaudi* AS-infected Swiss Webster mice sacrificed at GD/ED 10.**

1. Parasitemia in virgin and gravid Mal+ mice was estimated by flow cytometry and is presented as the percentage of iRBCs in the peripheral blood.
2. Percent hematocrit was measured throughout infection in virgin and gravid Mal+ mice and gravid Mal- controls.
3. Weight change in virgin and gravid Mal+ mice and gravid Mal- mice is presented as the percentage of body weight relative to 0 days post-infection or post-mock infection.
4. AUC was calculated for the parasitemia curve of each individual mouse. A statistically significant difference in parasite burdens is detected between virgin and gravid Mal+ mice (Student’s *t* test, *P* < 0.05).
5. AUC was calculated for the hematocrit curve of each individual mouse. Statistically significant differences in hematocrit are observed between virgin and gravid Mal+ mice (*P* < 0.001) and between gravid Mal- and Mal+ mice (*P* < 0.01; one-way ANOVA with Bonferroni multiple group comparisons).
6. AUC was calculated for the weight change curve of each individual mouse. A statistically significant differences in weight change is are observed between virgin and gravid Mal+ mice (*P* < 0.001) but no significant difference in weight change is observed between gravid Mal+ and Mal- mice (*P* > 0.05; one-way ANOVA with Bonferroni multiple group comparisons).

Virgin Mal+ *n* = 14; Gravid Mal+ *n* = 14; Gravid Mal- *n* = 11; Gravid Mal+, aborting *n* = 6; *** *P* ≤ 0.001; ** *P* ≤ 0.01; ns *P* > 0.05.

**
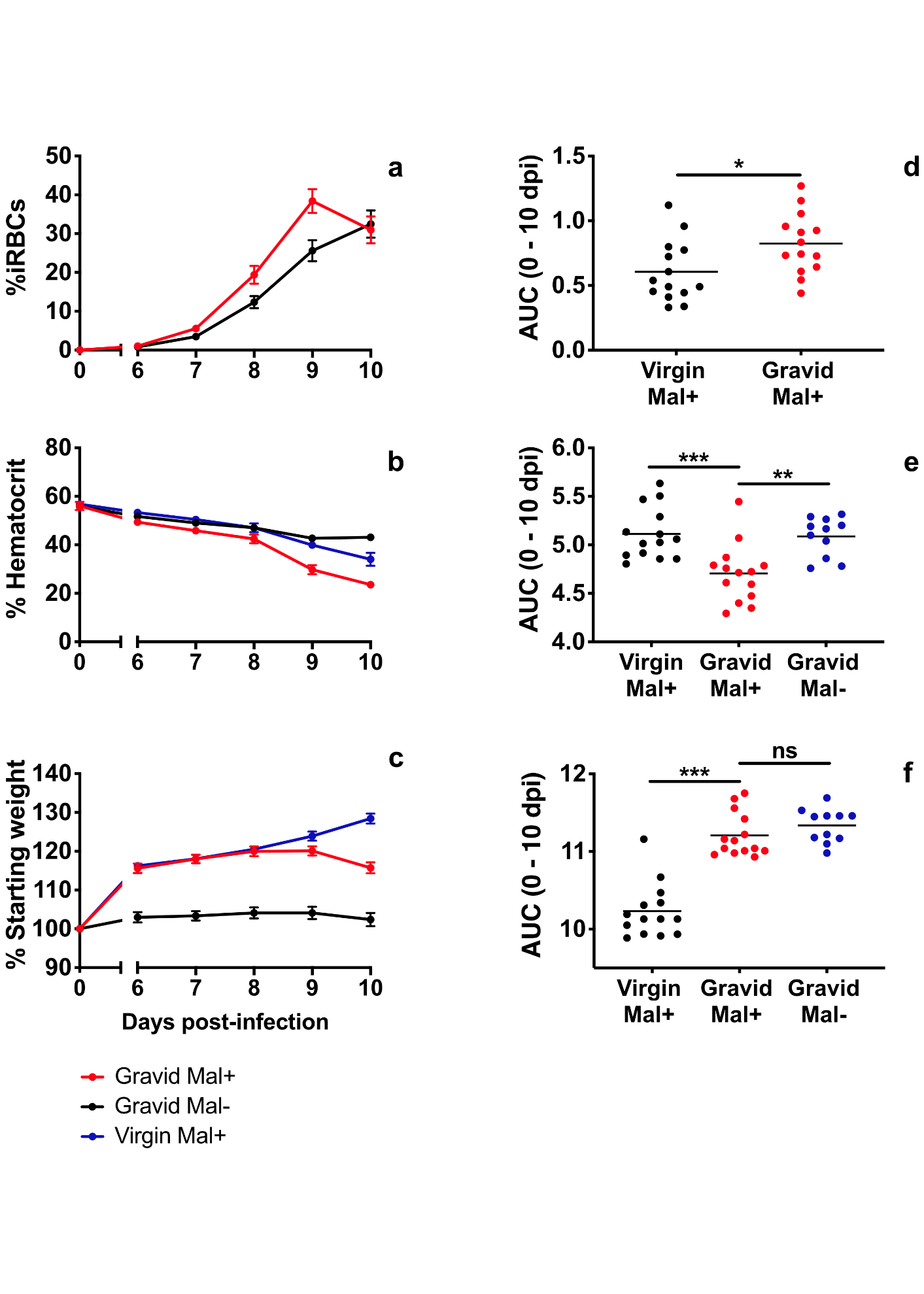
**

**Supplemental Figure 6. Peripheral and placental parasite burden in *P. chabaudi chabaudi* AS-infected dams at GD/ED 10.**

Paired peripheral and placental parasite burdens in Mal+ dams. No significant difference between peripheral and placental parasitemia is observed (*P* = 0.2402; Wilcoxon matched-pairs signed rank test). *n* = 11.

**Figure 7. Micrographs of placenta from Mal- mouse at GD/ED 10.**

(a-d) Placental junctional zone of two uninfected dams at GD 10 stained with hematoxylin and eosin. Trophoblastic erythrophagocytosis is evident (examples depicted with diamond arrows). Panels a and c were captured with a 60X objective; panels b and d are captured with a 100X objective from the same areas, respectively; bar = 20 um. MBS = maternal blood sinusoid.

**
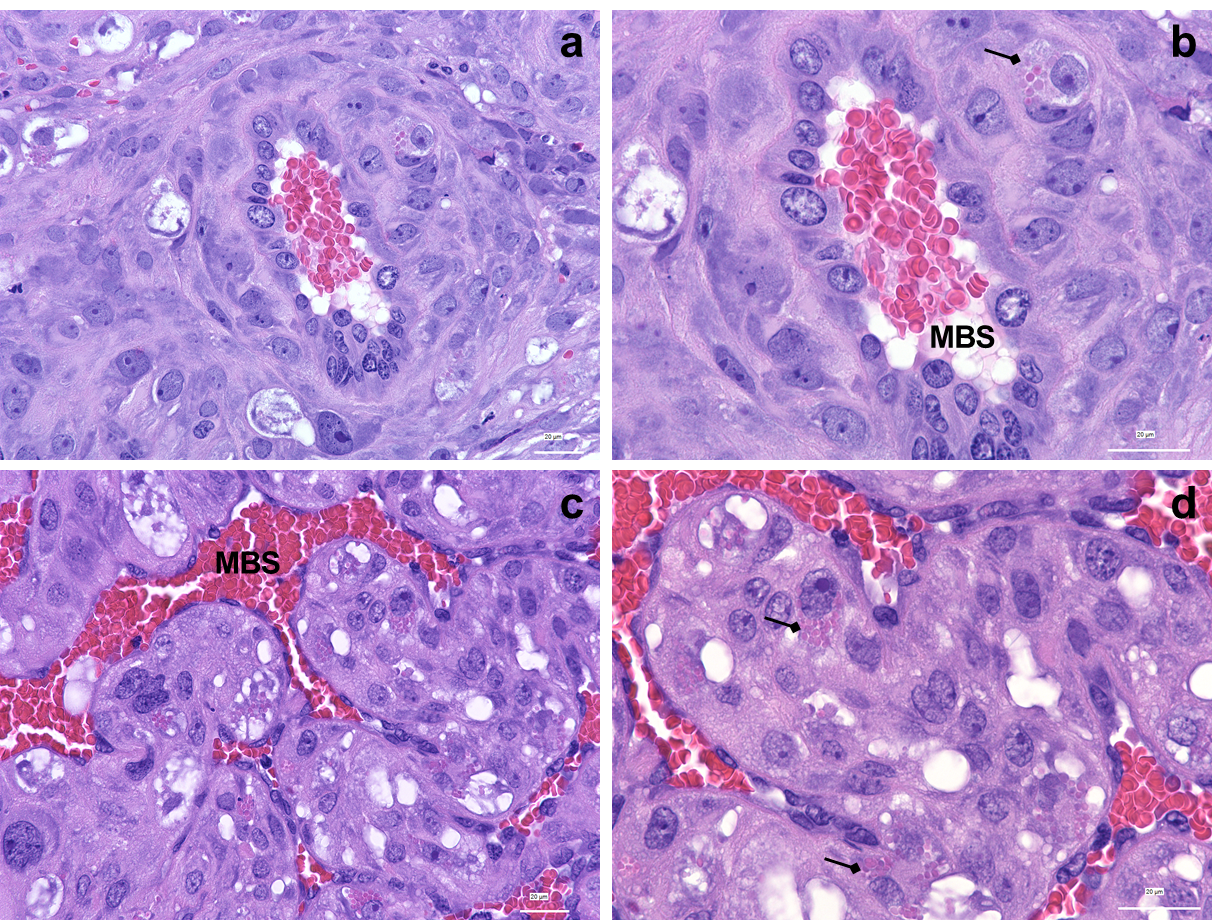
**

**Supplemental Table 1. Primer sequences.**

Mouse-specific forward (FP) and reverse (RP) primers used in real time PCR for the amplification of gene-of-interest and housekeeping (*Ubc*) transcripts and amplicon sizes are listed.

| **Gene** | **Forward and Reverse Primers** | **Amplicon size** |
| --- | --- | --- |
| *Ubc* | FP: 5’-CAGTGTTACCACCAAGAAGGT-3’ | 118 |
|  | RP: 5’-GAAAACTAAGACACCTCCCCCA-3’ |  |
| *Sod1* | FP: 5’-GGAACCATCCACTTCGAGCA-3’ | 135 |
|  | RP: 5’-CTGCACTGGTACAGCCTTGT-3’ |  |
| *Sod2* | FP: 5’-GGACCCATTGCAAGGAACAAC-3’ | 179 |
|  | RP: 5’-TGAGTGAGGTTTCACTTCTTGC-3’ |  |
| *Sod3* | FP: 5’-GCAACTCAGAGGCTCTTCCTC-3’ | 174 |
|  | RP: 5’-CCCCTGGATTTGACATGGTGA-3’ |  |
| *Cat* | FP: 5’-CACTGACGAGATGGCACACT-3’ | 174 |
|  | RP: 5’-TGTGGAGAATCGAACGGCAA-3’ |  |
| *Nrf2* | FP: 5’-AGCCAGCTGACCTCCTTAGA-3’ | 130 |
|  | RP: 5’-AGTGACTGACTGATGGCAGC-3’ |  |
| *Hmox1* | FP: 5’-CATAGCCCGGAGCCTGAATC-3’ | 146 |
|  | RP: 5’-AGCATTCTCGGCTTGGATGT-3’ |  |
